# Chronic Treatment with a Phytosomal Preparation Containing *Centella asiatica* L. and *Curcuma longa* L. Affects Local Protein Synthesis by Modulating the BDNF-mTOR-S6 Pathway

**DOI:** 10.3390/biomedicines8120544

**Published:** 2020-11-26

**Authors:** Giulia Sbrini, Paola Brivio, Enrico Sangiovanni, Marco Fumagalli, Giorgio Racagni, Mario Dell’Agli, Francesca Calabrese

**Affiliations:** Department of Pharmacological and Biomolecular Sciences, Università degli Studi di Milano, 20133 Milan, Italy; giulia.sbrini@unimi.it (G.S.); paola.brivio@unimi.it (P.B.); enrico.sangiovanni@unimi.it (E.S.); marco.fumagalli3@unimi.it (M.F.); giorgio.racagni@unimi.it (G.R.); mario.dellagli@unimi.it (M.D.)

**Keywords:** *Centella asiatica* L., *Curcuma longa* L., Bdnf, mTOR, protein synthesis, neuroplasticity, botanicals

## Abstract

Brain derived neurotrophic factor (Bdnf) is the most diffuse neurotrophin in the central nervous system and it is crucial for the proper brain development and maintenance. Indeed, through the binding to its high affinity receptor TRKB and the activation of different intracellular cascades, it boosts cell survival, neurite growth and spine maturations mechanisms. Here, we evaluated if the chronic oral treatment for 10 days with a phytosomal preparation containing *Centella asiatica* L. and *Curcuma longa* L. could improve Bdnf levels in the prefrontal cortex of adult rats. Interestingly we found an increased expression of Bdnf with main effect of the treatment on the mTOR-S6 downstream signaling pathway. Accordingly, we found an increase in the expression of eukaryotic elongation factor (eEF2) with a shift towards the phosphorylated form thus increasing the transcription of Oligophrenin-1, a protein carrying the upstream Open Reading Frame (uORF) which reduction is paralleled by memory dysfunctions. These results show the ability of the phytosome to enhance mTOR-S6 regulated transcription and suggest the possibility to use this preparation in subjects with impairments in neuroplastic mechanisms, memory and cognitive abilities.

## 1. Introduction

Brain-Derived Neurotrophic Factor (Bdnf) is the most diffuse neurotrophin, involved in several positive functions both during the development and at adulthood [1,2]. Indeed, it stimulates a plethora of mechanisms involved in cell survival, neurite growth and spine maturations through the activation of the high affinity receptor Tropomyosin Sensitive Receptor Kinase B (TRKB) that in turn activates different downstream pathways [3].

Among these intracellular mechanisms, Bdnf can stimulate protein translation by triggering the mammalian target of rapamycin (mTOR)-ribosomal protein (S6) signaling pathway thus boosting the activity of several translation-related proteins such as the initiation and the elongation factors [3,4]. Interestingly, although protein synthesis mainly occurs close to the nucleus, it can also happen at synaptic level improving neuroplasticity, memory and cognitive functions [5,6]. 

Protein synthesis is a complex process consisting of at least 3 steps: initiation, elongation and termination [7]. The ratio between the total and the phosphorylated form of the elements involved in the first two steps, namely eukaryotic initiation factors (eIF2) and eukaryotic elongation factors (eEF2), determines the set of proteins to be translated. In particular, when the total form is more abundant than the phosphorylated one, the general translation is preferred while it has been demonstrated that an increase of the phosphorylated form enhances the translation of peptides whose genes carry the upstream Open Reading Frame (uORF) [8,9,10]. 

Considering the positive effects of Bdnf, it is not surprising that its alterations are associated with different pathological conditions [11,12]. Hence, it has been demonstrated that different drugs and phytochemicals lead to beneficial effects at molecular levels and improve memory and cognitive functions by targeting Bdnf machinery [13,14,15,16,17,18,19]. 

However, the downstream mechanisms activated by Bdnf that may be responsible for the outcomes of both chemical and natural compounds are not completely defined. 

On these bases, the aim of this study was to determine the effect of the chronic administration for 10 days of a phytosomal preparation containing *Centella asiatica* L. (Gotu kola, Asiatic pennywort) and *Curcuma longa* L. (Turmeric) (50 mg/kg or 250 mg/kg) on Bdnf expression and on its high affinity receptor TRKB. Furthermore, we assessed whether the increase in Bdnf signaling was paralleled by an upregulation of de novo protein synthesis by focusing on the prefrontal cortex (PFC), a brain region affected by the phytosome ingredients [15,16].

## 2. Material and Methods

### 2.1. Plant Material

The Phytosome^®^ was provided by Indena S.p.A. and contained a purified and standardized dry extract from leaves of *Centella asiatica* L. (*C. asiatica*) (19.6%) and *Curcuma longa* L. (*C. longa*) rhizome extract (29.1% as total curcuminoid content). The percentage of *C. asiatica* L. asiaticoside in the phytosome formulation, analyzed by HPLC, was 3.8% according to ARM/79-8031. Phytosome is an innovative vesicular delivery system, where extracts are complexed with phosphatidylcholine, aimed at improving the absorption and the bioavailability of the active compounds. 

### 2.2. Animals

Adult male Sprague Dawley rats (Charles River, Calco, Italy) (10 weeks; 320–340 grams) were habituated to the laboratory conditions for one week before starting the experiment, and they were housed with food and water *ad libitum* with a 12 h light/dark cycle at constant temperature (22 ± 2 °C) and humidity (50 ± 5%) conditions.

All animal procedures were conducted according to the authorization n 977/2017-PR (approved on 11 December 2017) from the Italian Health Ministry in full accordance with the Italian legislation in animal experimentation (DL 26/2014) and conformed to EU recommendations (EEC Council Directive 2010/63). All efforts were made to minimize animal suffering and to reduce the number of animals.

### 2.3. Treatment

After the habituation, rats were chronically treated by oral gavage (os) once a day for 10 consecutive days with water (vehicle) or phytosomal preparation at 50 mg/kg or 250 mg/kg; composed by 20 mg/kg or 100 mg/kg of *C. asiatica* L. extract plus 30 mg/kg or 150 mg/kg of *C. longa* L., respectively. 

Two hours after the last administration, animals were anesthetized with isoflurane and then suppressed with CO_2_. After decapitation, the PFC, defined as cingulate cortex (Cg) 1–3 and infralimbic sub-regions (plates 6–10), was immediately dissected from 2 mm thick slices, according to the atlas of Paxinos and Watson [20] and then stored at −80 °C for the subsequent molecular analyses.

### 2.4. Quantification of Triterpenes and Curcuminoids in Plasma by LC-MS/MS Analysis

Curcumin and *C. asiatica* triterpenes were simultaneously quantified, in the plasma of rats treated with the phytosomal preparation containing *C. longa* and *C. asiatica*, by the LC–MS/MS method reported in Sbrini et al., 2020 [15]. Resveratrol was used as internal standard. Curcumin glucuronide, which is considered the most abundant curcumin metabolite in the blood, was also quantified. The analytes and the internal standard were quantified by using the following mass transitions: 367/149 (curcumin), 543/367 (curcumin glucuronide), 533/487 (asiatic acid), 549/503 (madecassic acid), 1003.8/958 (asiaticoside), and 1019.9/973 (madecassoside), 227/185 (resveratrol). 

### 2.5. RNA Preparation and Gene Expression Analysis by Quantitative Real-Time PCR

Total RNA was isolated by a single step of guanidinium isothiocyanate/phenol extraction by using a PureZol RNA isolation reagent (Bio-Rad Laboratories, Segrate, Italy) according to the manufacturer’s instructions and quantified by spectrophotometric analysis.

The samples were then processed for real-time polymerase chain reaction (RT-PCR) to assess total *Bdnf* and of *Bdnf* long 3′ untranslated region (UTR), (primer and probes sequences are listed in the Table 1). An aliquot of each sample was treated with DNase (Thermoscientific, Rodano, Italy) to avoid DNA contamination. RNA was analyzed by TaqMan qRT-PCR one-step RT-PCR kit for probes (Bio-Rad laboratories, Italy). Samples were run in 384 well formats in triplicate as multiplexed reactions with a normalizing internal control (*36b4*).

Thermal cycling was started with an incubation at 50 °C for 10 min (RNA retrotranscription) and then at 95 °C for 5 min (TaqMan polymerase activation). After this initial step, 39 cycles of PCR were performed. Each PCR cycle consisted of heating the samples at 95 °C for 10 s to enable the melting process and then for 30 s at 60 °C for the annealing and extension reactions. A comparative cycle threshold (Ct) method was used to calculate the relative target gene expression.

### 2.6. Protein Extraction and Western Blot Analysis

Western blot was employed to measure mature BDNF (mBDNF), TRKB (pTRKB Tyr816 and full-length), mammalian target of rapamycin (pmTOR Ser2448 and mTOR), ribosomal protein (pS6 Ser240/244 and S6), PLC (pPLC Tyr783 and PLC), AKT (pAKT Ser473 and AKT), ERK1 (pERK1 Thr202 and ERK1), ERK2 (pERK2 Tyr204 and ERK2), CAMP Responsive Element Binding Protein (pCREB Ser133 and CREB), eukaryotic initiation factor 2 (peIF2 Ser51and eIF2), eukaryotic elongation factor 2 (peEF2 Thr56 and eEF2) and Oligophrenin-1 (OPHN-1) protein levels in the crude synaptosomal fraction and in the whole homogenate (representative western blot bands of the proteins measured are showed in Figure S1).

Tissues were homogenized, and proteins were extracted as previously described [21]. The protein concentration of each sample was assessed according to the Bradford protein assay procedure (Bio-Rad Laboratories) with albumin (Sigma Aldrich, Milano, Italy) as the calibration standard. The purity of fraction was previously reported [22].

Western blot was run in reducing conditions by using Tris-Glycine eXtended (TGX) precast gel criterion (Bio-Rad Laboratories). All blots were blocked with 5% nonfat dried milk and incubated with the appropriate primary and secondary antibodies, as specified in Table 2.

Immunocomplexes were visualized with Western Lightning Clarity ECL (Bio-Rad Laboratories) and the Chemidoc MP imaging system (Bio-Rad Laboratories). Protein levels were quantified with ImageLab (Bio-Rad Laboratories) and normalized versus β-ACTIN.

### 2.7. Statistical Analysis

**Figure 1:**
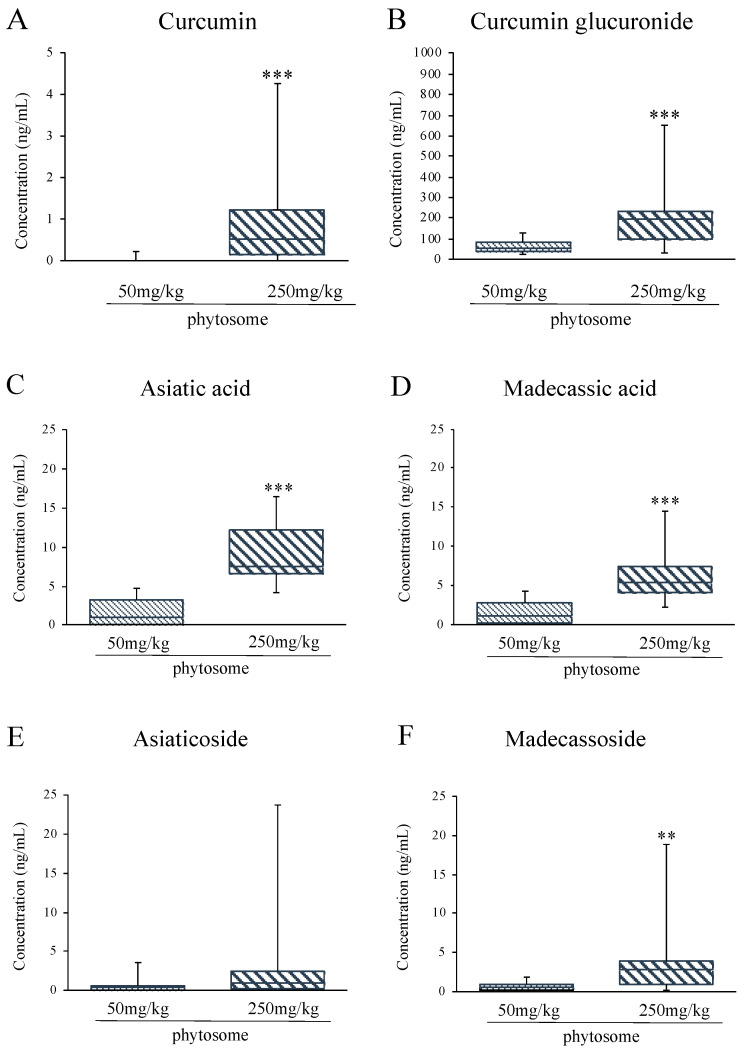
Concentration of Curcumin (**A**), Curcumin glucuronide (**B**), Asiatic acid (**C**), Madecassic acid (**D**), Asiaticoside (**E**) and Madecassoside (**F**) in the plasma of rats following the repeated oral administration of the phytosomal preparation. Data are expressed in ng/mL as are represented as boxes and whiskers graphs. ** *p* < 0.01, *** *p* < 0.001 vs. vehicle; Mann-Whitney nonparametric test.

All the data were checked for normality using the Kolmogorov-Smirnov test and the Shapiro-Wilk tests and for homoscedasticity with the Brown-Forsythe test and Bartlett’s test. Normally distributed and homoscedastic data were further analyzed using “IBM SPSS Statistics, version 26” with the one-way analysis of variance (ANOVA). When appropriate, further differences were analyzed by the Fisher’s protected least significance difference (PLSD) method. Moreover, non-parametric datasets, belonging to Figure 1, were analyzed with Mann-Whitney nonparametric test. Significance for all tests was assumed for *p* < 0.05.

Each experimental group consisted of 5–6 rats, and data are presented as mean ± standard error (SEM).

## 3. Results

### 3.1. Quantification of Terpenes and Curcuminoids in Plasma by LC-MS/MS Analysis

To assess the plasmatic concentrations of the main compounds or their metabolites following repeated oral administration, an analytical method to quantify simultaneously terpenes and curcuminoids was set up. All compounds quantified reached concentrations in the ng/mL order and, as expected, were more abundant in the plasma of rats treated with the higher dose (250 mg/kg). 

Curcumin was present mostly as glucuronide, reaching concentrations 200-fold higher than the corresponding free form (192 vs. 0.93 ng/mL, respectively) in rats treated with 250 mg/kg phytosome (Figure 1A,B).

Terpenes quantification showed that the acid forms (Figure 1C,D) were found to be more abundant than the corresponding glucosides (Figure 1E,F); asiatic acid reached the highest concentration (9.2 ng/mL, corresponding to 19 nM).

### 3.2. Phytosome Administration Increased Bdnf Levels in the PFC

**Figure 2:**
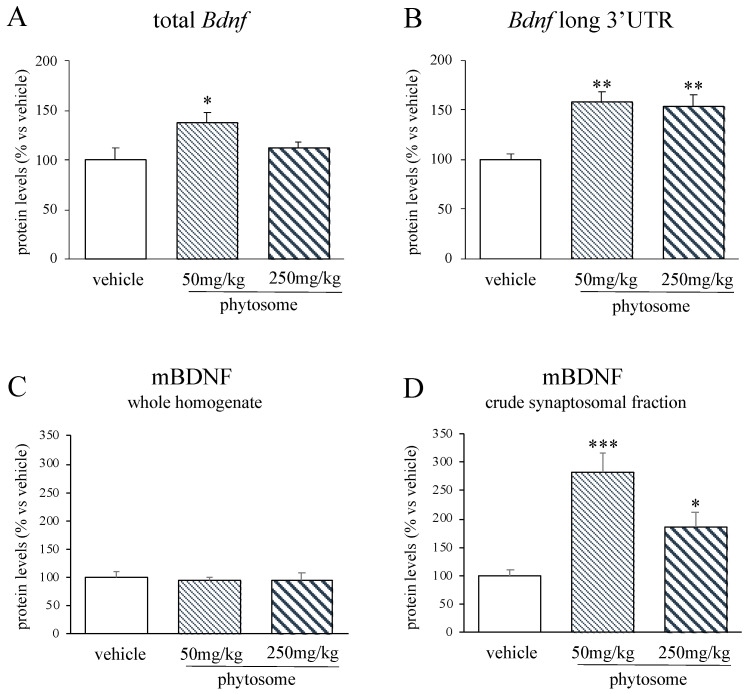
Analyses of total *Bdnf*, (**A**) *Bdnf* long 3′UTR (**B**) mRNA levels and mBDNF protein levels in the whole homogenate (**C**) and crude synaptosomal fraction (**D**) of the PFC of rats treated with phytosome 50 mg/kg or 250 mg/kg. Data are expressed as percent change of vehicle treated rats and are represented in the histograms graphs as mean ± SEM of 5–6 independent determinations. * *p* < 0.05, ** *p* < 0.01, *** *p* < 0.001 vs. vehicle; one-way ANOVA with Fisher’s PLSD.

Phytosome administration for 10 consecutive days significantly affected the expression of the neurotrophin *Bdnf* in the rat prefrontal cortex. As shown in Figure 2A, we observed a significant effect of the treatment (F_2-14_ = 4.085 *p* < 0.05, one-way ANOVA) on the total form of *Bdnf* with the lower dosage of phytosome that increased its mRNA levels (+38% *p* < 0.05 vs. vehicle, Fisher’s PLSD). Similarly, also the pool of the long transcripts of *Bdnf* was significantly modulated by the treatment (F_2-15_ = 11.245 *p* < 0.01, one-way ANOVA). Indeed, the administration of phytosome at both 50 and 250 mg/kg significantly up regulated its mRNA levels (+58% *p* < 0.01 vs. vehicle; +53% *p* < 0.01 vs. vehicle respectively; Fisher’s PLSD) (Figure 2B).

On the basis of the transcriptional results, we measured also the protein levels of the neurotrophin and accordingly we found that both the dosages induced a significant increase (50 mg/kg: +183% *p* < 0.001 vs. vehicle; 250 mg/kg: +87% *p* < 0.05 vs. vehicle; Fisher’s PLSD) in mBDNF protein levels in the crude synaptosomal fraction (F_2-14_ = 15.740 *p* < 0.001, one-way ANOVA) (Figure 2D). On the contrary, we did not observe any changes of mBDNF levels in the whole homogenate (Figure 2C).

### 3.3. The Increase of mBDNF Protein Levels Was Paralleled by an Increased Activity of Its Receptor TRKB

**Figure 3:**
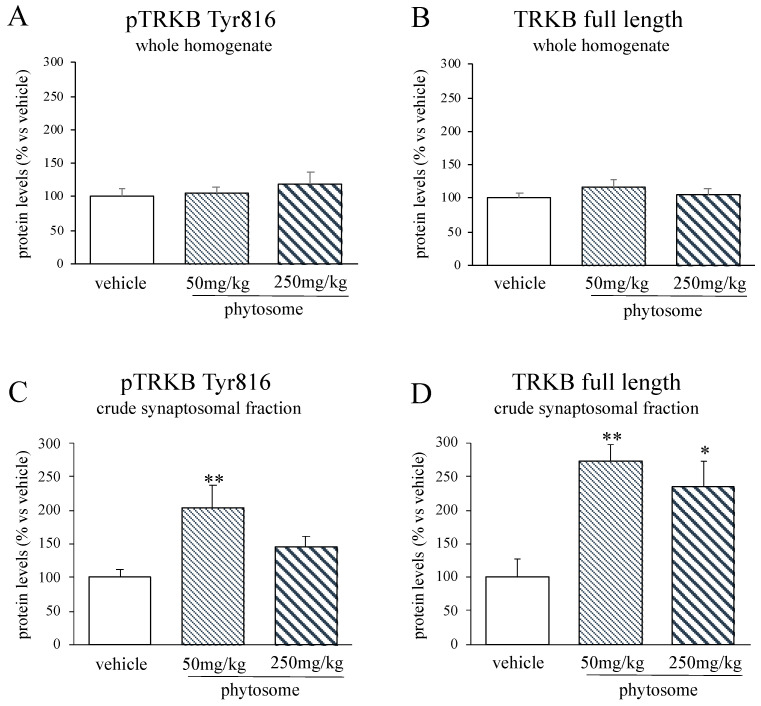
Analyses of pTRKB Tyr816 (**A**–**C**) and TRKB full length (**B**–**D**) protein levels in the whole homogenate (**A**,**B**) and crude synaptosomal fraction (**C**,**D**) of the PFC of rats treated with phytosome 50 mg/kg or 250 mg/kg. Data are expressed as percent change of vehicle treated rats and are represented in the histograms graphs as mean ± SEM of 4–6 independent determinations. * *p* < 0.05, ** *p* < 0.01 vs. vehicle; one-way ANOVA with Fisher’s PLSD.

In the whole homogenate, in line with our findings on mBDNF protein levels, we did not observe any changes neither in the phosphorylated form nor in the total levels of the receptor TRKB (Figure 3A,B), whereas in the crude synaptosomal fraction, phytosome administration positively affected both pTRKB Tyr816 and TRKB full length protein levels. Indeed, as revealed by the significant effect of the treatment (F_2-14_ = 5.873 *p* < 0.05, one-way ANOVA), the phosphorylated form was increased in the crude synaptosomal fraction of rats treated with the lower dose of phytosome (+103% vs. vehicle *p* < 0.01; Fisher’s PLSD), while we observed only a not significant increase with the higher dosage (+45% *p* > 0.05 vs. vehicle; Fisher’s PLSD) (Figure 3C). Similarly, we found a significant effect of the treatment on TRKB full length protein levels (F_2-12_ = 6.284 *p* < 0.05, one-way ANOVA), that were increased at both the dosages tested (50 mg/kg: +173% *p* < 0.01; 250 mg/kg: +135% *p* < 0.05 vs. vehicle; Fisher’s PLSD) (Figure 3D).

### 3.4. TRKB Phosphorylation in Phytosome-Treated Animals Specifically Activated mTOR-S6 Intracellular Signaling Pathway

The phosphorylation of the high affinity receptor TRKB produces the activation of different intracellular signaling cascades as result of several stimuli thus producing a specific outcome in term of brain plasticity [3].

**Figure 4:**
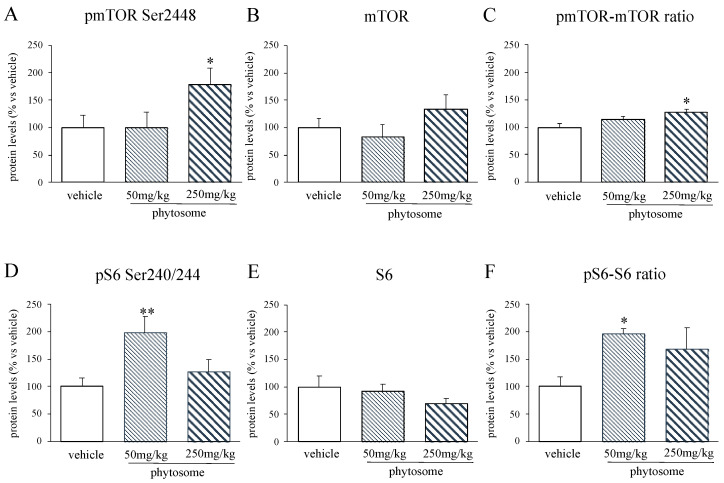
Analyses of mTOR (**A**–**C**) and S6 (**D**–**F**) protein levels in the PFC of rats treated with phytosome 50 mg/kg or 250 mg/kg. Data are expressed as percent change of vehicle treated rats and are represented in the histograms graphs as mean ± SEM of 3–6 independent determinations. * *p* < 0.05, ** *p* < 0.01 vs. vehicle; one-way ANOVA with Fisher’s PLSD.

In this study, we investigated some of these pathways uncovering that our experimental conditions specifically affected the mTOR-S6 signaling, which is well-known to control protein synthesis and cell growth [23]. In particular, the phosphorylated form of mTOR in Ser2448 was upregulated by the higher dose of phytosome (+79% *p* < 0.05 vs. vehicle; Fisher’s PLSD) (treatment effect: F_2-11_ = 3.016 *p* < 0.05, one-way ANOVA) whereas we did not found changes in total mTOR protein levels (Figure 4A,B). Therefore, as shown in Figure 4C, we observed a significant effect of the treatment on the ratio pmTOR/mTOR (F_2-11_ = 4.865 *p* < 0.05, one-way ANOVA), specifically in the animals treated with phytosome 250 mg/kg (+27% *p* < 0.05 vs. vehicle; Fisher’s PLSD).

Similarly, the active form of S6 (pS6 Ser240/244) was affected (Figure 4D), as indicated by the significant effect of the treatment (F_2-13_ = 5.202 *p* < 0.05, one-way ANOVA) with the lower dose, which significantly increased pS6 Ser240/244 protein levels (+97% *p* < 0.05 vs. vehicle; Fisher’s PLSD). On the contrary, no changes were found on the total form of S6 (Figure 4E).

Accordingly, we observed a significant increase of the ratio between the activated form of S6 and its total protein (F_2-13_ = 5.650 *p* < 0.05, one-way ANOVA) due to the treatments (phytosome 50 mg/kg: +97% *p* < 0.05; phytosome 250 mg/kg: +68% *p* > 0.05 vs. vehicle; Fisher’s PLSD) (Figure 4F).

Finally, the other pathways investigated were only partially modulated with changes restricted to pAKT Ser473 (treatment effect: F_2-13_ = 3.998 *p* < 0.05, one-way ANOVA), pAKT Ser473 and AKT ratio (treatment effect: F_2-14_ = 5.705 *p* < 0.05, one-way ANOVA) and pERK2 Tyr204 (treatment effect: F_2-14_ = 3.301 *p* > 0.05, one-way ANOVA) protein levels (results summarized in Table 3).

### 3.5. Local Protein Synthesis Is Boosted by Phytosome Administration

mTOR together with S6 contributes to the control of the translational machinery [24]. In particular, the phosphorylation of these two factors can trigger the translational initiation and the peptide elongation thus increasing the protein synthesis and contributing to neuronal plasticity [25,26].

Here, we observed no effect of the treatment on the initiation process with unchanged eIF2 protein levels (Table 3).

**Figure 5:**
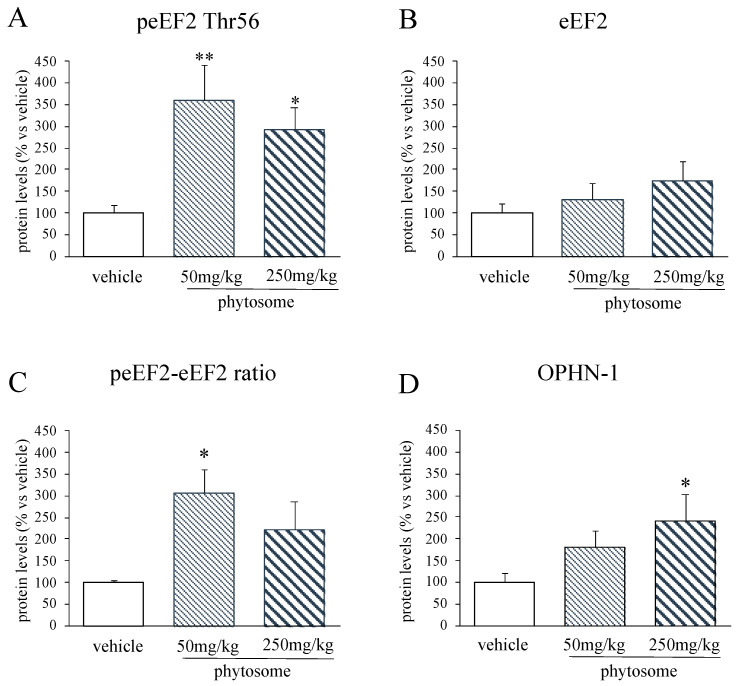
Analyses of eEF2 (**A**–**C**) and OPHN-1 (**D**) protein levels in the PFC of rats treated with phytosome 50 mg/kg or 250 mg/kg. Data are expressed as percent change of vehicle treated rats and are represented in the histograms graphs as mean ± SEM of 4–6 independent determinations. * *p* < 0.05, ** *p* < 0.01 vs. vehicle; one-way ANOVA with Fisher’s PLSD.

On the contrary, protein elongation process was modulated by phytosome administration. In particular, we observed a significant effect of the treatment on peEF2 Thr56 protein levels (F_2-14_ = 7.044 *p* < 0.01, one-way ANOVA) with both doses increasing its protein levels (50 mg/kg: +259% *p* < 0.01 vs. vehicle; 250 mg/kg: +194% *p* < 0.05 vs. vehicle; Fisher’s PLSD) (Figure 5A); however, no changes were observed for the total form of eEF2 (Figure 5B). Accordingly, the ratio peEF2/eEF2 (Figure 5C) was significantly increased in treated rats (50 mg/kg: +207% *p* < 0.05 vs. vehicle; 250 mg/kg: +123% *p* > 0.05 vs. vehicle; Fisher’s PLSD) (treatment effect: F_2-13_ = 4.944 *p* < 0.05, one-way ANOVA).

It has been demonstrated that a shift of the balance between the phosphorylated and the total form of eEF2 towards the phosphorylated form leads to an increased translation of specific mRNA containing the uORF sequence [10]; our results suggest that phytosome administration may enhance the synthesis of new proteins containing the uORF in their sequence.

On this bases, we measured the expression of OPHN-1 that contains 2 uORF and, as shown in Figure 5D, we observed an increase in the protein levels in rats treated with the lower dose (+81% *p* > 0.05 vs. vehicle; Fisher’s PLSD) and a significant up-regulation due to the higher dose administration (+143% *p* < 0.05 vs. vehicle; Fisher’s PLSD), despite the effect of the treatment was not statistically significant (F_2-11_ = 2.730 *p* > 0.05, one-way ANOVA).

## 4. Discussion

In the present study, we provide evidence that the chronic administration of the phytosomal preparation containing *C. asiatica* and *C. longa* positively affects neuroplastic mechanisms by increasing the expression of Bdnf and the activation of the downstream pathways via TRKB receptor. Moreover, we observed that the increase of the neurotrophin induced changes in the translation machinery with an enhanced activation of proteins involved in cognitive and memory processes.

In particular, we measured the neurotrophin expression in the PFC of phytosome-treated rats finding an increase of the total form of *Bdnf* and of the long 3′UTR pool of transcripts. Moreover, in line with the preferential localization of *Bdnf* mRNAs carrying the long 3′UTR in the dendrites where they are translated in case of demand [27], we observed an upregulation of mBDNF protein levels in the synaptic fraction. These findings suggest that 10 days of phytosome administration may promote neuroplastic mechanisms by increasing the production of trophic factors as, for example, mBDNF. Accordingly, a similar effect was observed after the administration of a wide range of botanicals as well as pharmacological pure compounds [14,15,16,17,18,19,28,29,30]. Interestingly, both the active principles contained in the phytosome (terpenes from *C. asiatica* and curcumin from *C. longa*) may contribute to the effects described in the present paper. Indeed, it has been previously reported that *C. asiatica* extract up-regulates Bdnf thus improving memory performance [15,17]. Moreover, Wang R. and colleagues showed in vitro protective effects of curcumin against glutamate excitotoxicity in rat cortical neurons, the effect was accompanied by an increase in the Bdnf level and activation of TRKB [18]. Similarly, in the same cell model, curcumin produced neuroprotective effects via mBDNF/TRKB-dependent MAPK and PI-3K cascades [19]; however, this is the first study investigating in vivo a combination of the two botanicals in a phytosomal formulation.

Furthermore, we demonstrated that the increase of mBDNF protein levels induced by the treatment was reflected by an increase of the phosphorylated and active form of TRKB receptor suggesting that the higher amount of mBDNF produced is released from the synapses, being able to activate its downstream pathways.

Notably, TRKB activation may set in motion different intracellular cascades [3,31] that can be specifically modulated by several compounds [32,33].

To deeper investigate the mechanism of action of the phytosomal preparation, we analyzed the activation of some pathways activated by an increased phosphorylation of the TRKB receptor finding the most relevant effect on mTOR-S6 signaling via AKT and ERK phosphorylation. Interestingly, ketamine, *Glycyrrhiza glabra* root extract as well as zinc administration induce a similar modulation on this pathway that is accompanied by an antidepressant-like effect [34,35], thus suggesting a possible antidepressant effect of the phytosomal preparation.

Finally, since protein translation is also controlled by mTOR complex [7] that in turn can be boosted by increased levels of the neurotrophin [3], we measured the initiation and the elongation factors protein levels, and we found an increase of peEF2 Thr56 with respect to the total form of the protein due to phytosome administration. Interestingly, this shift toward the phosphorylated form, able to increase the translation of specific proteins carrying the uORF sequence [36], was paralleled by increased levels of OPHN-1. Notably, memory and cognitive alterations not only in animal models of depression but also in humans with X-linked mental retardation are characterized by impairments in OPHN-1 transduction and translation [6,37] supporting the possibility that chronic phytosome administration, by increasing OPHN-1 translation, could induce an enhancement in brain mechanisms crucially involved in cognitive processes.

## 5. Conclusions

In conclusion, our data support the use of phytosome preparation in ameliorating brain plasticity through the activation of mBDNF-mTOR-S6 intracellular cascade and the transcription of specific proteins involved in memory processes in the PFC. Hence, despite the necessity of further experiments focused on evaluation of the animal behavior following phytosomal administration, this phytosomal preparation could be used as supporting therapy in subjects characterized by memory and cognitive disfunctions mainly related to alterations in frontal brain regions.

## Figures and Tables

**Table 1:** sequence of forward and reverse primers and probes used in the real-time polymerase chain reaction analyses and purchased from Eurofins MWG-Operon (Germany) (**a**) and from Life technologies, which does not disclose the sequences (**b**).

**(a) Gene**	**Forward Primer**	**Reverse Primer**	**Probe**
*36b4*	TTCCCACTGGCTGAAAAGGT	CGCAGCCGCAAATGC	AAGGCCTTCCTGGCCGATCCATC
Total *Bdnf*	AAGTCTGCATTACATTCCTCGA	GTTTTCTGAAAGAGGGACAGTTTAT	TGTGGTTTGTTGCCGTTGCCAAG
**(b) Gene**		**Accession Number**	**Assay ID**
*Bdnf* long 3′UTR		EF125675	Rn02531967_s1

**Table 2:** antibodies condition used in the western blot analyses. Over/Night (O/N); Room Temperature (RT); Milk (M); Bovine serum albumin (BSA).

Protein	Primary Antibody	Secondary Antibody
mBDNF (14 KDa)	1:1000 M 3% (Icosagen) 4° O/N	Anti-mouse 1:2000 M 3% 1 h RT
pTRKB Y816 (145 KDa)	1:1000 BSA 5% (Cell Signalling) 4°O/N	Anti-rabbit 1:1000 M 3% 1 h RT
TRKB full length (145 KDa)	1:750 BSA 5% (Cell Signalling) 4° O/N	Anti-rabbit 1:1000 M 3% 1 h RT
pmTOR Ser2448 (289 KDa)	1:1000 BSA 5% (Cell Signalling) 4° O/N	Anti-rabbit 1:5000 M 3% 1 h RT
mTOR (289 KDa)	1:1000 BSA 5% (Cell Signalling) 4° O/N	Anti-rabbit 1:5000 M 3% 1 h RT
pS6 Ser240/244 (32 KDa)	1:1000 BSA 5% (Cell Signalling) 4° O/N	Anti-rabbit 1:2000 M 3% 1 h RT
S6 (32 KDa)	1:1000 BSA 5% (Cell Signalling) 4° O/N	Anti-rabbit 1:1000 M 3% 1 h RT
pPLC Tyr783 (155 KDa)	1:1000 BSA 5% (Cell Signalling) 4° O/N	Anti-rabbit 1:1000 M 5%, 1 h RT
PLC (155 KDa)	1:1000 BSA 5% (Cell Signalling) 4° O/N	Anti-rabbit 1:1000 M 3% 1 h RT
pAKT Ser473 (60 KDa)	1:1000 BSA 5% (Cell Signalling) 4° O/N	Anti-rabbit 1:1000 M 3% 1 h RT
AKT (60 KDa)	1:1000 BSA 5% (Cell Signalling) 4° O/N	Anti-rabbit 1:1000 M 3% 1 h RT
pERK1 Thr202 (44 KDa)	1:1000 BSA 5% (Cell Signalling) 4° O/N	Anti-rabbit 1:2000 M 3% 1 h RT
ERK1 (44 KDa)	1:1000 BSA 5% (Santa Cruz Biotechnology) 4° O/N	Anti-rabbit 1:5000 M 3% 1 h RT
pERK2 Tyr204 (44 KDa)	1:1000 BSA 5% (Cell Signalling) 4° O/N	Anti-rabbit 1:2000 M 3% 1 h RT
ERK2 (44 KDa)	1:1000 BSA 5% (Santa Cruz Biotechnology) 4° O/N	Anti-rabbit 1:5000 M 3% 1 h RT
pCREB Ser133 (43 KDa)	1:1000 BSA 5% (Cell Signalling) 4° O/N	Anti-rabbit 1:5000 M 3% 1 h RT
CREB (43 KDa)	1:1000 BSA 5% (Cell Signalling) 4° O/N	Anti-rabbit 1:5000 M 3% 1 h RT
peIF2 Ser51 (38 kDa)	1:1000 BSA 5% (Cell Signalling) 4° O/N	Anti-rabbit 1:1000 M 3% 1 h RT
eIF2 (38 kDa)	1:1000 BSA 5% (Cell Signalling) 4° O/N	Anti-rabbit 1:1000 M 3% 1 h RT
peEF2 Thr56(95 KDa)	1:1000 BSA 5% (Cell Signalling) 4° O/N	Anti-rabbit 1:1000 M 3% 1 h RT
eEF2 (95 KDa)	1:1000 BSA 5% (Cell Signalling) 4° O/N	Anti-rabbit 1:1000 M 3% 1 h RT
OPHN-1 (91 KDa)	1:1000 BSA 5% (Santa Cruz) 4° O/N	Anti-mouse 1:1000 M 3% 1 h RT
β-ACTIN (43 KDa)	1:10,000 M 3% (Sigma-Aldrich) 45 min RT	Anti-mouse 1:10,000 M 3% 45 min RT

**Table 3:** Analyses of PLC, AKT, ERK1, ERK2, CREB, eIF2 (phosphorylated and total) protein levels in the PFC of rats treated with phytosome 50 mg/kg or 250 mg/kg. Data are expressed as percent change of vehicle treated rats and are expressed as mean ± SEM of 3–6 independent determinations. * *p* < 0.05 vs. vehicle; one-way ANOVA with Fisher’s PLSD.

Protein	Vehicle	Phytosome 50 mg/kg	Phytosome 250 mg/kg
pPLC Tyr783	100 ± 10	76 ± 7	104 ± 17
PLC	100 ± 13	97 ± 9	114 ± 15
pPLC–PLC ratio	100 ± 5	94 ± 7	92 ± 10
pAKT Ser473	100 ± 13	200 ± 35 *	203 ± 34 *
AKT	100 ± 28	115 ± 20	85 ± 5
pAKT–AKT ratio	100 ± 16	165 ± 64	204 ± 31 *
pERK1 Thr202	100 ± 21	96 ± 15	106 ± 24
ERK1	100 ± 15	101 ± 13	81 ± 11
pERK1–ERK1 ratio	100 ± 8	127 ± 22	131 ± 29
pERK2 Tyr204	100 ± 20	142 ± 21	202 ± 37 *
ERK2	100 ± 13	97 ± 7	80 ± 9
pERK2–ERK2 ratio	100 ± 46	110 ± 18	187 ± 49
pCREB Ser133	100 ± 18	115 ± 27	87 ± 15
CREB	100 ± 14	107 ± 19	114 ± 38
pCREB–CREB ratio	100 ± 10	131 ± 24	118 ± 21
peIF2 Ser51	100 ± 11	123 ± 23	118 ± 15
eIF2	100 ± 6	116 ± 15	111 ± 10
peIF2–eIF2 ratio	100 ± 10	104 ± 11	105 ± 9

## Supplementary Materials

The following are available online at https://www.mdpi.com/2227-9059/8/12/544/s1, Figure S1: Representative western blot bands of the proteins measured.
