# Supplementary figures and images for: Chronic Treatment with a Phytosomal Preparation Containing Centella asiatica L. and Curcuma longa L. Affects Local Protein Synthesis by Modulating the BDNF-mTOR-S6 Pathway
Source: Biomedicines. 2020 Nov 26;8(12):544. doi: 10.3390/biomedicines8120544 (PMC7761389; doi:10.3390/biomedicines8120544)

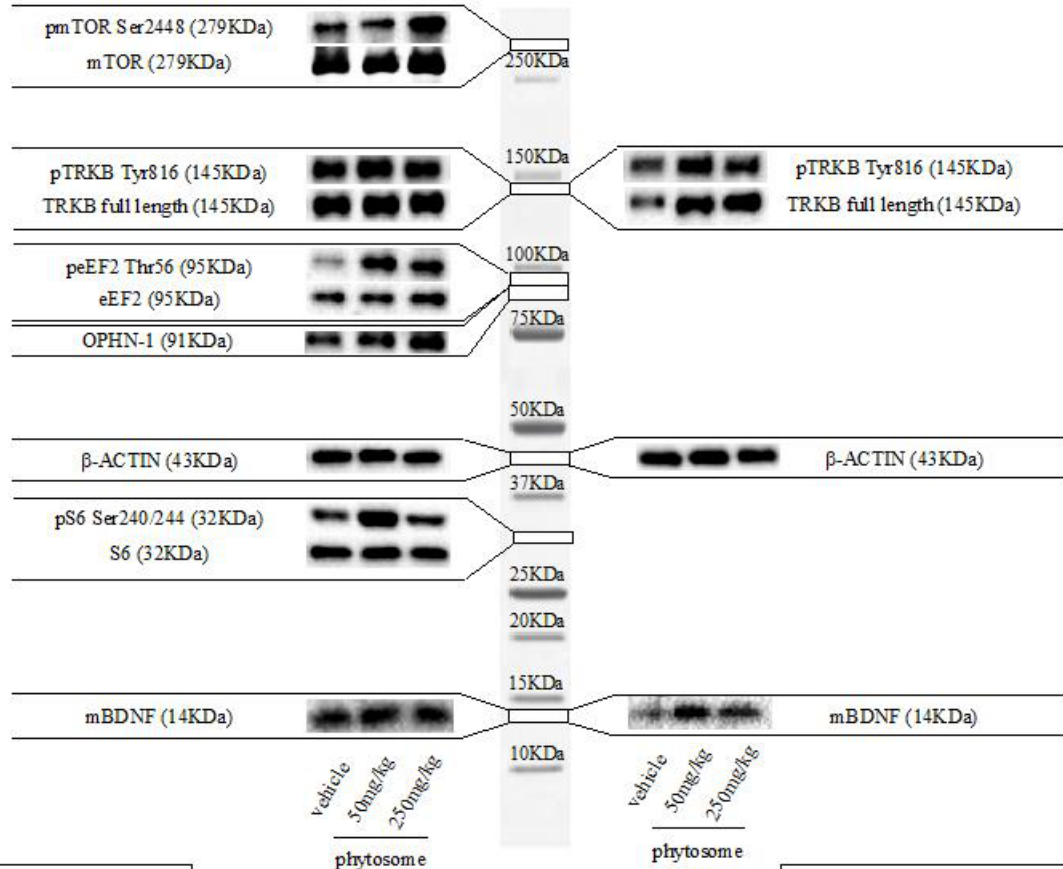

whole homogenate

crude synaptosomal fraction

Supplement: Supplementary file 1 [file biomedicines-08-00544-s001.pdf]
